# Regional Homogeneity within the Default Mode Network in Bipolar Depression: A Resting-State Functional Magnetic Resonance Imaging Study

**DOI:** 10.1371/journal.pone.0048181

**Published:** 2012-11-02

**Authors:** Chun-Hong Liu, Xin Ma, Feng Li, Yong-Jun Wang, Chang-Le Tie, Su-Fang Li, Tao-Lin Chen, Ting-ting Fan, Yu Zhang, Jie Dong, Li Yao, Xia Wu, Chuan-Yue Wang

**Affiliations:** 1 Department of Radiology, Beijing Anding Hospital, Capital Medical University, Beijing, China; 2 Beijing Key Lab of Mental Disorders, Beijing Anding Hospital, Capital Medical University, Beijing, China; 3 Center of the Treatment in Depressive Disorders, Beijing Anding Hospital, Capital Medical University, Beijing, China; 4 State Key Laboratory of Cognitive Neuroscience and Learning, Beijing Normal University, Beijing, China; 5 College of Information Science and Technology, Beijing Normal University, Beijing, China; 6 Beijing Key Laboratory of Brain Major Disorders - State Key Lab Incubation Base, Capital Medical University, Beijing, China; 7 Beijing Neuroscience Disciplines, Capital Medical University, Beijing, China; University of Cambridge, United Kingdom

## Abstract

**Aim:**

We sought to use a regional homogeneity (ReHo) approach as an index in resting-state functional magnetic resonance imaging (fMRI) to investigate the features of spontaneous brain activity within the default mode network (DMN) in patients suffering from bipolar depression (BD).

**Methods:**

Twenty-six patients with BD and 26 gender-, age-, and education-matched healthy subjects participated in the resting-state fMRI scans. We compared the differences in ReHo between the two groups within the DMN and investigated the relationships between sex, age, years of education, disease duration, the Hamilton Rating Scale for Depression (HAMD) total score, and ReHo in regions with significant group differences.

**Results:**

Our results revealed that bipolar depressed patients had increased ReHo in the left medial frontal gyrus and left inferior parietal lobe compared to healthy controls. No correlations were found between regional ReHo values and sex, age, and clinical features within the BD group.

**Conclusions:**

Our findings indicate that abnormal brain activity is mainly distributed within prefrontal-limbic circuits, which are believed to be involved in the pathophysiological mechanisms underlying bipolar depression.

## Introduction

Bipolar affective disorder is a severe, chronic, recurrent, and often lifetime psychiatric illness, and patients with this disease spend the majority of their time in episodes of depression as opposed to euthymia or mania [1], [2], [3]. Because bipolar depression (BD) is associated with suicide, as well as significant functional impairment, morbidity, and mortality, efforts should be made to identify the neurobiological mechanisms that contribute to the diathesis for this phase of the illness. In contrast to the mania and euthymia episodes, the existing findings concerning the underlying brain mechanism(s) of the depression episode of BD are far from complete [4]. Indeed, considering existing primary neuroimaging studies in BD patients using various tasks, Chen et al. (2011) pointed out that no single region has been consistently reported by more than two primary studies [4]. Recent research has suggested that brain activity in the resting-state reflects the baseline status of the brain and provides a promising aspect to investigate the pathophysiological characteristics of mental disorders (for reviews, see [5], [6]).

Investigations from various groups suggest that an abnormality of the default mode network (DMN) may play a critical role in the neural circuitry mediating BD [7], [8]. The DMN is a large-scale brain network that encompasses a specific set of brain regions, including the ventral medial prefrontal cortex, dorsal medial prefrontal cortex, posterior cingulate cortex/precuneus, ventral anterior cingulate cortex, lateral temporal cortex, hippocampus and surrounding cortex (e.g., parahippocampal cortex), and the medial, lateral, and inferior parietal lobe [9], [10], [11], [12]. Structural magnetic resonance imaging (sMRI) studies have compared bipolar disorder patients in multiple episodes relative to healthy controls (HC) and report rather heterogeneous findings within above DMN brain areas, including atrophy in the prefrontal cortex [13], [14], [15], the frontal lobe [14], the cingulate cortex [16], [17], and the hippocampus [18], as well as enlargement of the amygdala and temporal gyrus [14], [19]. Existing studies using resting-state functional magnetic resonance imaging (fMRI) also document that bipolar disorder is related to activation of the DMN [20], [21], [22]. In addition, a recent meta-analysis provides evidence for functional and anatomical alterations in bipolar disorder, suggesting that an imbalance between cortical cognitive and limbic-related brain networks may serve as a neurobiological marker of bipolar disorder [23]. According to above studies, there is strong evidence that an abnormality of the DMN might also play a critical role in the neural circuitry mediating bipolar depression [7], [8]. Despite the increasing knowledge of bipolar disorder, however, very little is known regarding resting-state regional brain activity within the DMN in depressed bipolar disorder relative to HC.

In the present study, we employed the regional homogeneity (ReHo) [24] approach to directly compare the resting-state brain activity between BD patients and HC subjects. Compared to the traditional seed-voxel approach as used in [22], the ReHo method focuses on the similarities or coherence of intraregional spontaneous low-frequency (<0.08 Hz) activity of the blood oxygenation level-dependent (BOLD) signal, which enables a novel perspective to understand the functional deficits in particular brain regions and provides more compatible information for integrating previous structural findings [24]. It may be potentially helpful to understand the level of coordination of regional neural activity in the resting-state and may be useful for revealing pathophysiological changes in human brain function [25], [26], [27]. As a piece of supporting evidence, Yan et al. (2010) revealed increased ReHo in the hippocampus of indigenous residents at Qinghai-Tibetan plateau under prolonged hypoxia exposure, suggesting that increased ReHo may be associated with the possible increase synchronizing ability in relevant regional neuronal activities as a mechanism to increase the blood supply to cope with chronic hypoxia [28]. This method has been widely applied to investigate many mental disorders, such as attention-deficit hyperactivity disorder (ADHD) [29], [30], [31], depression [32], [33], [34], [35], [36], autism spectrum diseases [37], schizophrenia [38], [39], Parkinson’s disease [27], and Alzheimer’s dementia [25].

In the current study, we aimed to explore the difference(s) within the DMN in the local functional connectivity, as reflected by ReHo, between BD patients and HC groups. We hypothesized that the local resting-state functional connectivity within the emotion-regulating circuit in the DMN would shed light on the pathogenesis of BD.

## Results

### The DMN Maps Determined by Group ICA

**Figure 1:**
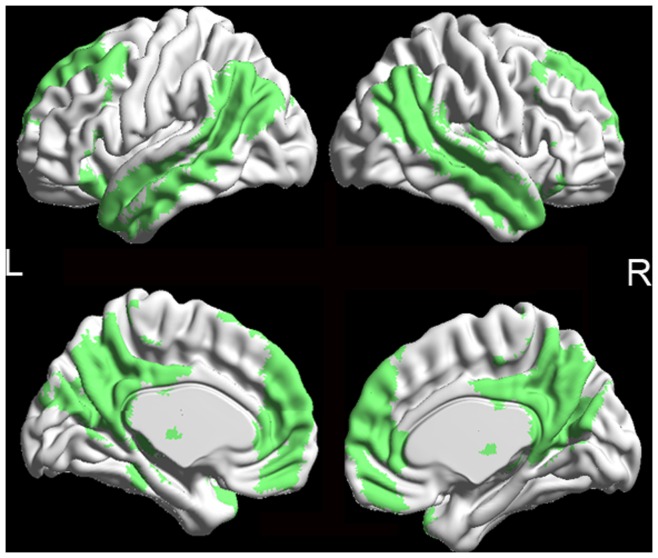
The DMN maps in both the BD and HC groups. These maps are the results of the within group one-sample *t*-test with family wise error (FWE) corrected *p* = 0.05.

Figure 1 shows the DMN in both the BD and HC groups, as determined by the group ICA approach. The DMN consists of the bilateral medial prefrontal cortex, bilateral middle frontal gyrus, ventral anterior cingulate cortex, posterior cingulate cortex/precuneus, lateral temporal cortex, hippocampus and surrounding cortex (e.g., parahippocampal cortex), and the medial, lateral, and inferior parietal lobe.

### Two-sample t-tests between the BD and HC Groups Within the DMN

**Figure 2:**
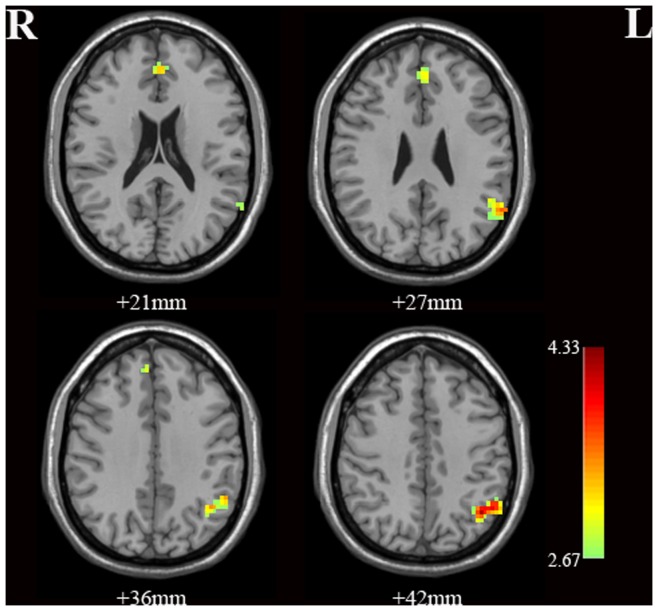
Statistical maps showing two sample *t*-test results of the ReHo maps between the BD and the HC groups (*p*<0.01, cluster size >12 voxels). Red and green denote increased ReHo. The color bars indicate the *t*-values.

Two-sample *t*-tests revealed significant differences between the two groups in two brain areas within the DMN (Table 1 and Figure 2). Specifically, the BD group displayed significantly increased ReHo in the following locations: the left medial frontal gyrus and the left inferior parietal lobe.

**Table 1:** Brain Areas with Significantly Increased Reho in Patients with BD Relative to the HCs^a^.

		Montreal Neurological	
		Institute Coordinate	Analysis
Extent of Cluster	Cluster Size (voxels)	Voxel (x,y,z)^b^	*t*
Left medial frontal gyrus	45	0, 42, 24	3.66
Left inferior parietal lobe	171	−42, −60, 48	5.28

a Differences were significant at *p* = 0.01.

b Data indicate coordinates corresponding to voxels with maximum (peak) effect sizes.

To perform a correlation analysis between altered ReHo values and clinical measurements, the average ReHo values of all voxels within the above two regions were separately extracted. Both the left medial frontal gyrus and left inferior parietal lobe displayed marginally significant (0.05<*p*<0.10) correlation with the number of depressive episodes. No brain region demonstrated significant correlations with sex, patient age, educational years, HAMD score, or illness length within the BD group (Table 2).

**Table 2:** Brain areas with significant between-group ReHo differences within the DMN and various clinical measures.

Brain regions	Side	Sex	Age	Education level	HAMD score	Number of depressive episodes	Illness length
Median frontal gyrus	Left	0.322	−0.156	−0.083	−0.065	0.378	0.217
Inferior parietal lobe	Left	0.149	0.134	−0.272	−0.011	0.350	0.042

Abbreviations: HAMD, Hamilton Depression Rating Scale. The values in the table are Pearson’s Correlation Coefficients.

## Discussion

Here, using BOLD resting-state fMRI and the ReHo analytical method, we found abnormal brain activity in the BD group relative to the HC group in several brain regions within the DMN. Significantly increased ReHo in the BD group was mainly found in the left medial frontal gyrus and the left inferior parietal lobe. To our knowledge, this is the first study of the DMN in bipolar depression using the ReHo method.

It is worthwhile to analyze the local connectivity of the time series within a functional cluster, and ReHo reflects intrinsic coherent neuronal activity within spatially organized brain regions [27]. Increased ReHo may be relevant to reflect neural hyperactivity in a regional brain area and vice versa [24]. The medial frontal gyrus, the hub of the DMN, is important for the ability of the affective value of reinforcers, decision making, and expectation [40]. In the current study, ReHo was significantly increased in the left medial frontal gyrus in the resting-state in BD patients, which reflects the enhancement of the local synchronization of spontaneous neural activities in this region. ReHo abnormalities observed in this region may be relevant to high ability in bipolar disorder [41] and support our understanding of the findings of prefrontal overactivity in bipolar disorder during up- and down-regulation of negative affect [42]. Conversely, several previous resting-state functional neuroimaging studies have found decreased ReHo in the medial frontal gyrus in other psychiatric disorders, including social anxiety disorder [43], heroin-dependent individuals [44], major depressive disorder [33], and schizophrenia [39]. Moreover, Lai et al. (2011) demonstrated that first-episode drug-naïve major depressive disorder with panic disorder patients displayed increased ReHo in the medial frontal cortex after short-term duloxetine therapy [45]. This suggests that ReHo differences in the medial frontal gyrus regions may demonstrate differences in the neurobiological substrates between bipolar disorder and other psychiatric disorders or secondary to medication. Future studies examining first-episode drug-naïve and different mood state bipolar disorder participants will aid in the clarification of the mechanisms behind increased homogeneity in bipolar patients.

The medial frontal gyrus is involved in both emotion perception and cognitive regulation functions [4], [21], [46], [47], and overactivity in this region may be responsible for the cognitive-emotional interference seen in BD. The abnormality of the medial frontal gyrus in BD patients has been reported in studies employing both emotional and cognitive tasks [4], [48], [49], [50]. Fusar-Poli et al. (2012) performed a meta-analysis of different tasks used in fMRI studies of individuals at enhanced genetic risk for bipolar disorder and found that an increased neural response exists for several regions, including the left superior frontal gyrus, medial frontal gyrus, and left insula [47]. In the current study, we found increased ReHo in the left medial frontal gyrus in the BD group, which indicates that there is baseline brain activity impairment in BD patients, supplementing the existing knowledge revealed by various cognitive and emotional tasks. Moreover, Osuch et al. (2000) find a direct correlation between depression severity and regional cerebral metabolism in the bilateral medial frontal gyrus in mood disorders, including bipolar patients [51]. In this study, we found the left medial frontal gyrus was marginally related to the number of depressive episodes. However, we did not find correlations between ReHo values and HAMD scores in this brain region, but this negative finding may be due to the narrow range of depression scores in our subjects. This suggests that the abnormal ReHo in the left medial frontal gyrus may be a biomarker, either trait or state marker, which is related to the depression episode of bipolar disorder. Further studies are required to verify this speculation.

Chepenik et al. (2010) demonstrated a decreased negative correlation between the activity of the left ventral prefrontal cortex and the amygdala in bipolar disorder subjects [22]. A noticeable difference between our current findings, e.g., in the left inferior parietal lobe, and those reported by Chepenik et al., (2010) concerns the amygdala. The medial frontal gyrus area is a functional hub that is closely connected to the ventral lateral prefrontal cortex and subcortical networks (e.g., limbic systems including the amygdala and frontoparietal networks) [21], [52], and converging neuroimaging evidence demonstrates the critical role of the medial frontal gyrus in emotional recognition and cognitive control [53], [54], [55]. Because local and global functional connectivity reveals the functional links (usually revealed by correlations) between a pair of regions within a region and the whole brain, respectively, we speculate that the impaired functional connectivity within the amygdala and inferior parietal lobe may due to dysfunction in the ventral prefrontal gyrus, especially the medial frontal gyrus, as found in the current study. Therefore, our findings do not conflict with or replicate existing functional connectivity studies. Instead, combining previous functional connectivity data and ReHo may increase our understanding of the impaired prefrontal limbic-related network underlying the neurobiology of bipolar disorder.

There are several limitations of the current study. First, the evaluation of the effects of medication is problematic in fMRI studies of medicated bipolar patients because the complete lifetime medication data (e.g., dose and duration) of the patients were difficult to obtain. Moreover, medications were not withdrawn at the time of the study due to ethical reasons [56]. Second, comparing BD patients with healthy controls cannot disentangle the differences between depressed and euthymic subjects from the differences between subjects with bipolar disorder and unaffected individuals. Given these limitations, future studies that include larger numbers of non-medicated subjects who are better balanced for age and take all of the above factors into account are warranted. In addition, to further clarify whether the ReHo abnormalities are shared by bipolar disorder patients in both depressed and euthymic episodes, future studies can compare patients in different episodes of BD with healthy controls.

In summary, we adopted ReHo to investigate the differences in resting-state brain activity between BD patients and healthy control subjects within the DMN. Our findings support a model of BD that involves dysfunction within prefrontal-limbic circuits, which may shed light on the pathophysiological mechanisms underlying BD.

## Methods

### Ethics Statement

The study protocol was approved by the Institutional Review Board of Beijing Anding Hospital, Capital Medical University, and Beijing Normal University Imaging Center for Brain Research. Each subject provided written informed consent.

### Participants

The demographic and clinical data are presented in Table 3. The subjects in the BD group underwent the Structured Clinical Interview for the Diagnostic and Statistical Manual of Mental Disorders (DSM-IV) (SCID) [57], which was locally validated, to obtain an accurate diagnosis from two experienced psychiatrists (Dr Feng Li and Dr Yong-Jun Wang). Both of the psychiatrists completed a 2-week training program prior to the diagnostic assessment [58], [59]. The inter-rater reliability of the SCID was tested and yielded satisfactory agreement. Inclusion criteria and for BD patients were as follows: 1) aged 18–60 years and ability to provide voluntary informed consent; 2) satisfied DSM-IV (SCID) criteria for bipolar disorder, were currently depressed (Young Mania Rating Scale (YMRS) score ≤7) [60] or in a depressive episode (17-item Hamilton Rating Scale for Depression (HAMD) [61] score ≥17), and had no history of schizophrenia, obsessive–compulsive disorder, or an anxiety disorder; 3) satisfied criteria to undergo an MRI scan based on an MRI screening questionnaire; and 4) were able to be managed as outpatients. All of our patients received normal brain MRI scans during the study to exclude the influence of other concomitant disorders. Twenty-six age-, sex-, and education-matched healthy subjects were recruited from the local area by poster advertisement and were excluded if they reported a first-degree relative with a mood disorder or for any exclusion criteria for MRI scanning. The Nonpatient Version Structured Interview from the DSM-IV was used to screen the healthy subjects to confirm the absence of a history of psychiatric or neurologic illness. All subjects were right-handed as measured by the Edinburgh Inventory [62].

**Table 3:** Demographic and clinical characteristics of the participants.

Variables (Mean±S.D.)	Bipolar depression(n = 26)	Healthy controls(n = 26)	*Statistics*	*p-*value
Gender (M: F)Age (years)	9∶1732.35±11.31	10∶1631.92±12.19	*x* ^2^ = 0.083T(1, 50) = 0.132	0.770.90
Age range	20–57	20–58	_	–
Education level (years)	12.32±2.0	13.15±2.13	T(1, 50) = 1.449	0.15
HAMD score	19.65±2.48	–	–	–
Duration of disease (years)	4.20±1.70	–	–	–
Number of depressive episodes	3.44±1.45	–	–	–
On Antidepressants		–	–	–
Citalopram	7	–	–	–
Sertraline	8	–	–	–
On Mood-stabilizer		–	–	–
Lithium	19	–	–	–
Sodium valproate	6	–	–	–
Divalproex	15	–	–	–
On Antipsychotics	16	–	–	–
Quetiapin	10	–	–	–
Olanzapin	1	–	–	–
Risperidone	5	–	–	–

Abbreviations: S. D., standard deviation; HAMD, Hamilton Depression Rating Scale.

### Data Acquisition

MR images were acquired on a 3.0 Tesla MR scanner (Magnetom Trio, Siemens, Erlangen, Germany). Each subject lay supine with their head snugly fixed by a belt and foam pads. The resting-state fMRI scanning sessions (including 33 axial slices, TR = 2000 ms, TE = 30 ms, FA = 90°, thickness/gap = 3.5/0.6 mm, in-plane resolution = 64×64, field of view (FOV) = 220×220 mm, matrix = 64×64, and 240 volumes) lasted 8 min. During the scan, subjects were instructed to relax with their eyes closed but not to fall asleep, which was later confirmed with a survey including the questions: 1) Did you fall asleep during the scanning?; 2) Did you keep both eyes closed during the scanning?; and 3) What were you thinking about during the scanning? The 3D-T1 session covered the entire brain: 128 sagittal slices, TR = 2530 ms, TE = 3.39 ms, slice thickness/gap = 1.33/0 mm, in-plane resolution = 256×192, inversion time (TI) = 1100 ms, FOV = 256×256 mm, and flip angle = 7°.

### Data Analyses

All the participants displayed no abnormalities on the conventional MRI assessed by two experienced radiologists (Dr. Yu Zhang and Dr. Jie Dong). All data were analyzed using REST software (http://resting-fmri.sourceforge.net) and SPM8 (http://www.fil.ion.ucl.ac.uk/spm). The first 10 time points were discarded due to scanner calibration and adaptation of the subject to the circumstances, leaving 230 time points for the preprocessing steps of slice timing, head motion correction, and spatial normalization with SPM8. We calculated the maximum excursion movement values for each of the translation planes (x, y, and z) and each of the rotation planes (roll, pitch, and yaw) for every participant. None of the participants had >2 mm maximum displacement in three planes or 2° angular motion during the entire fMRI scan. The 3D-T1 images were also spatially normalized to the Montreal Neurological Institute (MNI) template. Following this step, all images spatially normalized to the MNI template were then transformed into Talairach and Tournoux coordinates, and several sources of spurious variances (including the estimated motion parameters, linear drift, and global average BOLD signals) were removed from the data through linear regression [9], [63]. It is reported that the respiratory frequency (0.1–0.5 Hz) and aliased cardiac frequency range (0.6–1.2 Hz) are relatively high [64]. Zuo et al., [65] also reported that low frequency oscillations (0.01–0.073 Hz) are primarily detected within the gray matter, and relatively high frequency oscillations (0.073–0.25 Hz) are primarily restricted to the white matter. Thus, band pass filtering (e.g., 0.01–0.08 Hz) was used for further ReHo analysis using REST software [65]. ReHo results were smoothed with a Gaussian kernel of full width at half maximum (FWHM) of 4 mm. A Kendall’s correlation coefficient (KCC) value (also called the ReHo value) was calculated to measure the similarity of the time series of a given voxel to its nearest 26 voxels. The formula to calculate the KCC value is explained in previous studies [24], [37] and calculated as follows:
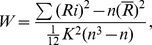
where W is the KCC value of that voxel, Ri is the sum rank of the i^th^ time point; 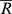 =  ((n +1) K)/2 is the mean of the Ri’s, K is the number of voxels within a measured cluster (K can be chosen among 7, 19, and 27, i.e., the number of nearest neighbors plus that given voxel), and n is the number for the ranks (i.e., the number for fMRI data volumes). Zang and colleagues [24] calculated the KCC value of every voxel in the brain, generating individual ReHo maps. Then, a mask (made from the MNI template to assure matching with the normalization step) was used to remove non-brain tissues and noise from the ReHo maps. To reduce the influence of individual variations in the KCC value, normalization of the ReHo maps was performed by dividing the KCC among each voxel by the averaged KCC of the entire brain within the mask [31], [66]. The fMRI toolbox GIFT (http://mialab.mrn.org/software/#gica) was used to perform the group independent component analysis (ICA) to select the DMN as a mask. There are three main stages of group ICA: data reduction, independent component separation, and back reconstruction. The outputs from these stages are multiple time courses, and every time course has an associated image map. The number of independent components was estimated from the fMRI data using the minimum description length (MDL) criteria. We obtained the common ICs and the corresponding time courses over all subjects after ICA separation using the Infomax algorithm, and back reconstruction of the ICs was performed based on the above results. Each subject component and time course were converted into z-scores. The independent components of the DMN were selected in both of the experimental groups. We selected the DMN according to several previous studies [67], [68]. Using this group ICA analysis, the DMN that we delineated was divided into two parts: the anterior DMN (aDMN) and posterior DMN (pDMN) [69].

### Statistical Analyses

One-sample *t*-tests were performed with family wise error (FWE) corrected (*p* = 0.05) for group ICA to identify the DMN (Figure 1). To explore the ReHo differences between the BD group and healthy control group, a second-level, random-effect, two-sample *t*-test was performed on the individual normalized ReHo maps in a voxel-by-voxel manner within the DMN. Although gender and age were not significantly different between groups, it was included as a covariate to avoid any undetected gender and age effects. Voxels with *p*<0.01 (corrected) and a cluster size >486 mm^3^ (12 voxels) were considered to show significant differences between the two groups. We then extracted the mean ReHo values of the clusters that differed between the two groups. Linear correlations were calculated between the mean ReHo values across all voxels in the abnormal areas in the BD group and clinical (age, sex, and educational years, illness length, number of depressive episodes, and HAMD score) variables.
